# Retraction: Titanium dioxide nanoparticles induce mitochondria-associated apoptosis in HepG2 cells

**DOI:** 10.1039/d5ra90014j

**Published:** 2025-02-19

**Authors:** Zhenglin Xia, Jingliang He, Bowei Li, Ke He, Wenbing Yang, Xiaoxun Chen, Jinqian Zhang, Guoan Xiang

**Affiliations:** a Department of General Surgery, Guangdong Second Provincial General Hospital, Southern Medical University Guangzhou 510515 People’s Republic of China guoan_66@163.com; b Shunde Hospital of Guangzhou University of Chinese Medicine Foshan 528300 People’s Republic of China; c Department of General Surgery, The Second Affiliated Hospital of Xi’an Jiao Tong University Xi’an 710004 People’s Republic of China; d Department of Gastrointestinal Surgery, The Guigang City People’s Hospital Guigang Guangxi 537100 People’s Republic of China; e Department of Laboratory Medicine, Guangdong Second Provincial General Hospital, Southern Medical University Guangzhou 510515 People’s Republic of China jingwanghou@163.com

## Abstract

Retraction of ‘Titanium dioxide nanoparticles induce mitochondria-associated apoptosis in HepG2 cells’ by Zhenglin Xia *et al.*, *RSC Adv*., 2018, **8**, 31764–31776, https://doi.org/10.1039/C8RA05132A.

The Royal Society of Chemistry hereby wholly retracts this *RSC Advances* article due to concerns with the reliability of the data.

There are similarities in the western blot data of the blots for α-ENaC in Fig. 12a of this article and the left-hand five blots for caspase-3 in Fig. 4a in ref. 1.

There are similarities in the western blot data of the blots for GAPDH in Fig. 12b of this article and the left-hand five blots for GAPDH in Fig. 4a in ref. 1, the left-hand five blots for GAPDH in Fig. 4c in ref. 1, and the first four blots for GAPDH in Fig. 2f and in Fig. 5c of ref. 2.

The authors were unable to provide complete raw data for this article, so we are unable to validate the integrity of this data. Given the significance of the concerns about the validity of the data, and the lack of raw data, the findings presented in this paper are not reliable.

All authors were informed about the retraction of the article. Zhenglin Xia agrees with the decision to retract this article, the other authors have not responded.

Signed: Zhenglin Xia

Date: 20th January 2025

Retraction endorsed by Laura Fisher, Executive Editor, *RSC Advances*

**References**

1 Z. Xia, X. Huang, K. Chen, H. Wang, J. Xiao, K. He, R. Huang, X. Duan, H. Liu, J. Zhang and G. Xiang, Proapoptotic Role of Potassium Ions in Liver Cells, *Biomed Res. Int.*, 2016, **2016**, 1729135, DOI: 10.1155/2016/1729135.

2 M. Zhang, J. Zhang, S. Liu, W. Qi, G. Lin, R. Qiu, M. Quan and J. Cheng, NS5ATP9 Suppresses Activation of Human Hepatic Stellate Cells, Possibly via Inhibition of Smad3/Phosphorylated-Smad3 Expression, *Inflammation*, 2015, **38**, 278–289.

